# Cumulative Reproductive Outcomes Across Three Embryo Transfer Cycles After Hysteroscopic Endometrial Polypectomy Using a Tissue Removal System in Infertile Women: A Single-Center Retrospective Cohort Study

**DOI:** 10.3390/diagnostics16091386

**Published:** 2026-05-02

**Authors:** Yurie Nako, Kiyotaka Kawai, Shoko Katsumata, Yuko Takayanagi, Shogo Nishii, Tatsuyuki Ogawa, Makiko Tajima, Osamu Hiraike

**Affiliations:** 1 Department of Reproductive Medicine, Kameda IVF Clinic Makuhari, Chiba 261-8501, Japan; kawaikiyotaka@wfc-mom.jp (K.K.);; 2 Department of Reproductive Medicine, Omotesando ART Clinic, Tokyo 107-0061, Japan; 3 Department of Obstetrics and Gynecology, Nippon Medical School Hospital, Tokyo 113-8603, Japan; osamuwh-tky@umin.ac.jp

**Keywords:** endometrial polyp, hysteroscopic tissue removal system, embryo transfer, endometrial polypectomy, infertility

## Abstract

**Background/Objectives:** This study aimed to describe cumulative reproductive outcomes across three embryo transfer (ET) cycles after hysteroscopic endometrial polypectomy using a hysteroscopic tissue removal system (HTRS) and to identify determinants of cumulative and per-cycle pregnancy. **Methods:** In this single-center retrospective cohort study, we included infertile women who underwent HTRS-based endometrial polypectomy between January 2023 and December 2024 and subsequently initiated at least one ET cycle. Patients were followed from ET1 through ET3. The primary endpoint was the cumulative clinical pregnancy rate (CCPR) within three ET cycles. In the observed cumulative analysis, treatment discontinuation was considered as non-pregnancy. Kaplan–Meier (KM) analysis was used to estimate the cumulative pregnancy probability, with treatment discontinuation considered as censoring. Multivariate logistic regression and generalized estimating equations were used to identify patient-level and cycle-level predictors. **Results:** Among 100 patients, 79 achieved clinical pregnancy within three ET cycles (CCPR 79.0%). The KM estimate at ET3 was 87.4%, and the cumulative live birth rate was 65.0%. Pregnancy rates declined with advancing maternal age (≤34 years, 91.9%; 35–39 years, 78.3%; ≥40 years, 52.9%). Maternal age independently predicted lower cumulative pregnancy and lower per-cycle pregnancy probability, whereas blastocyst transfer was associated with a higher probability of pregnancy per cycle. **Conclusions:** In women who underwent ET after HTRS polypectomy, cumulative pregnancy across three ET cycles was relatively high; however progression to live birth declined with advancing maternal age. As no non-surgical comparison group was included, these findings should be interpreted as descriptive rather than causal.

## 1. Introduction

Endometrial polyps are among the most common intrauterine abnormalities identified in infertile women, with a reported prevalence of 32–35% [1,2]. These benign lesions may impair embryo implantation via mechanical obstruction, induction of local inflammation, alteration of cytokine expression, and disruption of endometrial receptivity [3,4,5]. In assisted reproductive technology, even subtle intrauterine abnormalities can have important clinical implications because they may reduce implantation potential, lead to repeated unsuccessful embryo transfer (ET), prolong treatment duration, and increase the physical, emotional, and financial burden on patients. Therefore, appropriate diagnosis and treatment of endometrial polyps before ET are important components of optimizing the uterine environment in infertile women.

Hysteroscopic polypectomy is the standard treatment for endometrial polyps. Traditionally, resectoscopic electrosurgical resection and blind dilation and curettage have been performed. However, electrosurgery can lead to thermal injury to the basal endometrium and intrauterine adhesions, whereas blind curettage can cause incomplete removal [6,7]. These concerns are particularly relevant in infertile women planning ET, in whom preservation of the endometrial basal layer and complete removal of intracavitary lesions are clinically important.

Hysteroscopic tissue removal systems (HTRS), such as MyoSure and TruClear, are increasingly used because of their ability to mechanically excise lesions without thermal damage under continuous visualization. Randomized controlled trials have shown shorter operative times, higher completeness of resection, and fewer complications than traditional resectoscopic approaches [8,9,10,11]. These technical advantages provide a rationale for focusing specifically on HTRS-assisted polypectomy rather than hysteroscopic polypectomy in general. However, although HTRS may be theoretically favorable for endometrial preservation, its reproductive impact after subsequent ET remains insufficiently characterized, particularly when outcomes are evaluated cumulatively over repeated transfer attempts.

Several studies have examined reproductive and pregnancy outcomes after polypectomy, but data on cumulative outcomes across multiple ET cycles remain limited [6,12,13,14]. Single-cycle pregnancy rates provide useful information regarding the probability of success in an individual transfer; however, cumulative pregnancy probability is often more clinically meaningful in assisted reproduction because patients frequently undergo more than one ET cycle before achieving pregnancy [15]. In addition, treatment discontinuation before completing planned ET cycles may influence the interpretation of cumulative outcomes, especially in women of advanced reproductive age. Therefore, this study aimed to describe the cumulative clinical pregnancy rates (CCPR) for up to three ET cycles after HTRS-assisted hysteroscopic endometrial polypectomy in infertile women and to evaluate factors associated with patient- and cycle-level pregnancy outcomes.

## 2. Materials and Methods

### 2.1. Study Design and Setting

This retrospective observational study included all consecutive infertile women who underwent hysteroscopic endometrial polypectomy using HTRS at a single fertility center between January 2023 and December 2024.

### 2.2. Participants

The inclusion criterion was the initiation of at least one ET after surgery. Patients were followed up until pregnancy, treatment discontinuation, or completion of ET3.

A total of 121 consecutive infertile women who underwent hysteroscopic endometrial polypectomy using HTRS were initially assessed for eligibility. Of these, 21 were excluded because ET was not initiated after surgery (spontaneous pregnancy or intrauterine insemination, *n* = 8; treatment discontinuation, *n* = 10; submucosal leiomyoma, *n* = 1; atypical polypoid adenomyoma, *n* = 1; donor oocyte cycle, *n* = 1).

The final study cohort comprised 100 patients who underwent at least one ET after hysteroscopic polypectomy and were included in the primary patient-level analysis.

### 2.3. Ethical Approval and Consent

The study was conducted in accordance with the Declaration of Helsinki and approved by the Institutional Review Board of Kameda IVF Clinic Makuhari (protocol code WFC26-005; date of approval 5 March 2026). This retrospective chart review used clinical data collected during routine care between January 2023 and December 2024. The research protocol, including the secondary use of existing medical records and the opt-out consent procedure, was reviewed and approved by the institutional ethics committee.

Patients were informed through the institutional website and were allowed to decline participation.

### 2.4. Anesthesia and Hysteroscopic Surgical Procedures

The procedures were performed under intravenous sedation with propofol and paracervical block. Patients with cervical stenosis underwent preoperative dilation (Lamiken R; Kaneka Corp., Osaka, Japan). Normal saline was used as the distension medium. The lesions were excised under direct visualization using MyoSure Manual and MyoSure LITE (Hologic, Inc., Marlborough, MA, USA), or TruClear (Medtronic, Minneapolis, MN, USA). Device selection was based on lesion size and location. Operative time was defined as the time from hysteroscope insertion to removal. Thermal energy was not used in this study.

### 2.5. Chronic Endometritis (CE) Assessment

Endometrial samples collected during polypectomy were stained with CD138. Plasma cells were counted in 10 non-overlapping high-power fields (×400). CE scoring: 0 (0 plasma cells/10 HPF), 1 (1–5 plasma cells/10 HPF), 2 (6–15 plasma cells/10 HPF), and 3 (≥16 plasma cells/10 HPF).

Patients with a CE score ≥2 received antibiotics (doxycycline 200 mg/day or metronidazole 1000 mg/day for 1–2 weeks) before ET. Repeat biopsies were not performed routinely.

### 2.6. Embryo Transfer Protocol

ET was performed during the first menstrual cycle following surgery. The protocols included natural-cycle frozen–thawed ET, hormone replacement frozen–thawed ET, and fresh ET. Embryos were evaluated using the Veeck criteria for cleavage-stage embryos and the Gardner criteria for blastocysts. Good-quality blastocysts were defined as AA, AB, or BA. In double ET cycles, the two best-available embryos were preferentially selected for transfer. For analyses involving blastocyst quality, cycle-level embryo quality was assigned according to the highest-grade embryo transferred; thus, double ET cycles were classified as having a good-quality blastocyst when at least one transferred blastocyst was graded AA, AB, or BA. Endometrial thickness was measured on the day of ET. Preimplantation genetic testing for aneuploidy (PGT–A) was not performed.

Among the patients who did not achieve clinical pregnancy after ET1 (*n* = 49), nine discontinued treatment before ET2 (no further visits, *n* = 1; transfer to another clinic, *n* = 1; oocyte depletion, *n* = 7). Among patients who did not achieve clinical pregnancy after ET2 (*n* = 18), four discontinued treatment before ET3 (requested discontinuation, *n* = 1; oocyte depletion, *n* = 3).

### 2.7. Outcome Measures

The primary outcome was CCPR within the first three ET cycles (ET1–ET3). Clinical pregnancy was defined as visualization of an intrauterine gestational sac using transvaginal ultrasonography.

The cumulative live birth rate (CLBR) within three ET cycles was evaluated as a key secondary outcome. Live birth was defined as the delivery of a viable infant at >22 weeks of gestation.

CCPR was defined as the occurrence of a clinical pregnancy within three ET cycles after hysteroscopic polypectomy. Patients who failed to achieve clinical pregnancy within three ET cycles, including those who discontinued treatment before completing the three transfers, were classified as having no pregnancy in the observed cumulative analysis. In addition, Kaplan–Meier estimation (KM) was performed to assess cumulative pregnancy probability over time, in which clinical pregnancy was treated as the event of interest and treatment discontinuation was treated as right censoring.

### 2.8. Statistical Analysis

Continuous variables are presented as mean ± standard deviation (SD), and categorical variables are presented as numbers (%). Group comparisons were performed using Student’s *t*-test or one-way analysis of variance (ANOVA) for continuous variables and the chi-square test or Fisher’s exact test for categorical variables, as appropriate.

#### 2.8.1. Primary Analysis: Patient-Level Cumulative Pregnancy

The primary patient-level analysis evaluated factors associated with cumulative clinical pregnancy within the first three ET cycles (ET1–ET3). For descriptive purposes, the observed CCPR and CLBR within three ET cycles were calculated as proportions among all patients who initiated ET1. In this observed cumulative analysis, patients who discontinued treatment before completing three ET cycles without achieving clinical pregnancy were classified as non-pregnant.

To estimate cumulative pregnancy probability while accounting for treatment discontinuation, KM analysis was additionally performed, with clinical pregnancy treated as the event of interest and treatment discontinuation without pregnancy treated as right censoring. The number of ET cycles was used as a time scale. Age-stratified cumulative pregnancy curves were compared using the log-rank test, and Cox proportional hazards regression was performed to estimate hazard ratios (HRs) and 95% confidence intervals (CIs) according to maternal age group (≤34, 35–39, and ≥40 years), with the ≤34 years group used as the reference category.

To identify independent predictors of cumulative clinical pregnancy, multivariable logistic regression was performed using the binary outcome of at least one clinical pregnancy within up to three ET cycles. Given the 79 pregnancy events, the multivariable model was restricted to six prespecified covariates to maintain an event-per-variable ratio of approximately 10 and to reduce overfitting. The model included maternal age (per year), body mass index (BMI), number of endometrial polyps (1, 2–4, and ≥5; reference = 1), CE score (per-unit increase), endometriosis, and male infertility. Adjusted odds ratios (ORs) with 95% CIs are reported.

#### 2.8.2. Secondary Analysis: Cycle-Level Pregnancy Probability

A secondary cycle-level analysis was conducted to evaluate factors associated with the probability of clinical pregnancy in each ET cycle. Each ET cycle was treated as a single observation. The cycle-level dataset included all ET cycles performed up to ET3, including re-entry cycles after pregnancy loss, yielding a total of 162 cycles.

Multiple ET cycles could arise from the same patient; therefore generalized estimating equations (GEEs) with a binomial distribution and logit link were used to account for within-patient clustering. An exchangeable working correlation structure and robust (sandwich) standard errors were applied to the data. The dependent variable was clinical pregnancy in each ET cycle.

To reduce conceptual overlap among embryo-related predictors, embryo stage and blastocyst quality were not entered together as co-primary predictors in the same main all-cycle model, because blastocyst quality can only be defined among blastocyst transfer cycles. Therefore, the primary all-cycle GEE model included maternal age, embryo stage (blastocyst vs. cleavage-stage), number of embryos transferred, and endometrial thickness measured on the day of ET.

A sensitivity analysis was additionally performed, restricting the dataset to blastocyst transfer cycles only. In this blastocyst-only GEE model, maternal age, number of embryos transferred, endometrial thickness measured on the day of ET, and the presence of at least one good-quality blastocyst transferred were included as covariates. In double ET cycles, the blastocyst quality variable was coded according to the higher-grade embryo transferred; thus, cycles were classified as positive when at least one transferred blastocyst was graded as AA, AB, or BA.

Potential multicollinearity among candidate covariates was assessed using variance inflation factors (VIFs) based on the corresponding conventional logistic regression models. All VIF values were close to 1.0, indicating no evidence of multicollinearity. Population-averaged ORs with 95% CIs are reported. All statistical tests were two-sided, and *p* < 0.05 was considered statistically significant. Statistical analyses were performed using R version 4.3.1 (R Foundation for Statistical Computing, Vienna, Austria) and EZR version 1.68 (Saitama Medical Center, Jichi Medical University, Saitama, Japan).

Supplementary Materials supporting this study are publicly available at Zenodo (https://doi.org/10.5281/zenodo.19652688, Published 15 March 2026).

## 3. Results

### 3.1. Study Population and Baseline Characteristics

**Figure 1:**
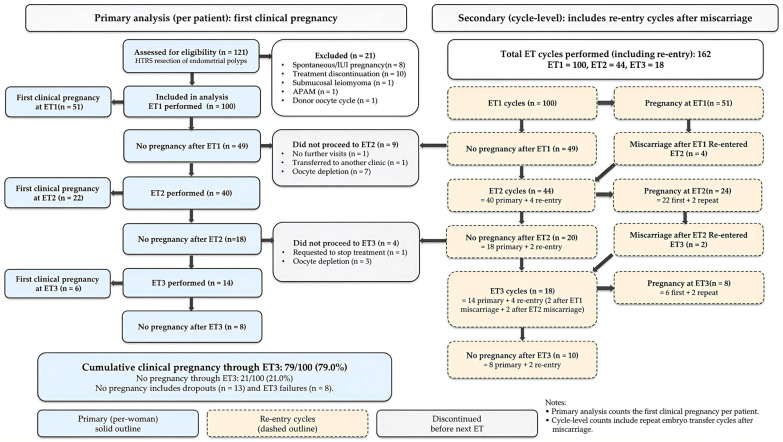
Flow diagram of embryo transfer cycles (ET1–ET3) and re-entry cycles after miscarriage. The left panel presents the primary per-patient analysis of the first clinical pregnancy within up to three ET cycles after HTRS polypectomy, in which patients who discontinued treatment before completing three cycles without pregnancy were classified as non-pregnant. The right panel presents the secondary cycle-level dataset, including re-entry cycles after miscarriage, resulting in 162 ET cycles in total (ET1 = 100, ET2 = 44, ET3 = 18). Clinical pregnancy was defined as ultrasonographic visualization of a gestational sac. ET, embryo transfer; HTRS, hysteroscopic tissue removal system; APAM, atypical polypoid adenomyoma; CCPR, cumulative clinical pregnancy rate.

A flowchart of the study is shown in Figure 1. Of the 121 women assessed for eligibility after hysteroscopic endometrial polypectomy using HTRS, 21 were excluded, and 100 who initiated ET1 were included in the primary (per-woman) analysis. Importantly, among the eight patients who achieved pregnancy before initiating ET, four conceived spontaneously and four achieved pregnancy following intrauterine insemination. These patients were excluded from the primary analysis because this study aimed to evaluate the cumulative reproductive outcomes among patients who underwent ET after surgery.

For the secondary cycle-level analyses, all ET cycles performed up to ET3, including re-entry cycles after miscarriage, were included (162 ET cycles: ET1 = 100, ET2 = 44, and ET3 = 18).

The baseline characteristics of the patients are summarized in Table 1. Among the 100 patients included, 79 achieved clinical pregnancy within three ET cycles, whereas 21 did not, including those who discontinued treatment before completing three cycles.

Patients who achieved pregnancy were significantly younger (35.08 ± 3.45 vs. 38.28 ± 3.19 years, *p* < 0.001) and had a lower BMI (22.10 ± 3.08 vs. 23.95 ± 4.23 kg/m^2^, *p* = 0.03). Gravidity, parity, anti-Müllerian hormone levels, and the number of preoperative ET cycles did not differ significantly between the groups. The distribution of polyp numbers differed between the groups, but the difference was not statistically significant (*p* = 0.05). CE scores and infertility etiologies were comparable between the groups.

### 3.2. Embryo-Transfer Characteristics (Cycle-Level Analysis)

The cycle-level ET characteristics are presented in Table 2. In this analysis, each ET cycle was treated as an independent observation, and all cycles performed up to the third transfer were included in the analysis. The total number of cycles exceeded the number of patients because pregnancy status was defined at the patient level.

A total of 121 and 41 ET cycles were performed in the pregnancy and non-pregnancy groups, respectively. The mean number of embryos transferred per cycle was significantly lower in the pregnancy group than in the non-pregnancy group (1.23 ± 0.42 vs. 1.41 ± 0.50, *p* = 0.02). Single ET was more frequently performed in the pregnancy group (93 vs. 24 cycles), whereas double ET was more common in the non-pregnancy group (*p* = 0.02).

Endometrial thickness at the time of transfer did not differ significantly between the groups (11.11 ± 2.05 mm vs. 10.61 ± 1.76 mm, *p* = 0.16). Similarly, the distribution of fresh and frozen–thawed ET cycles was comparable (*p* = 0.44).

Cleavage-stage ET cycles were significantly more frequent in the non-pregnancy group than in the pregnancy group (7 vs. 5 cycles, *p* = 0.01). Although blastocyst transfer cycles were more common in the pregnancy group (116 vs. 34 cycles), cycles with at least one good-quality blastocyst transferred were also more frequent in the pregnancy group than in the non-pregnancy group (90 vs. 17 cycles, *p* = 0.004).

### 3.3. Surgical Characteristics

The surgical characteristics were comparable between the two groups. The distribution of HTRS devices (MyoSure Manual, MyoSure LITE, and TruClear) did not differ significantly according to cumulative pregnancy outcome (*p* = 1.00).

### 3.4. Cumulative Clinical Pregnancy Rates and Survival Analysis

All 100 patients initiated ET1 and were included in the cumulative analyses.

#### 3.4.1. Observed Cumulative Clinical Pregnancy Rate

**Figure 2:**
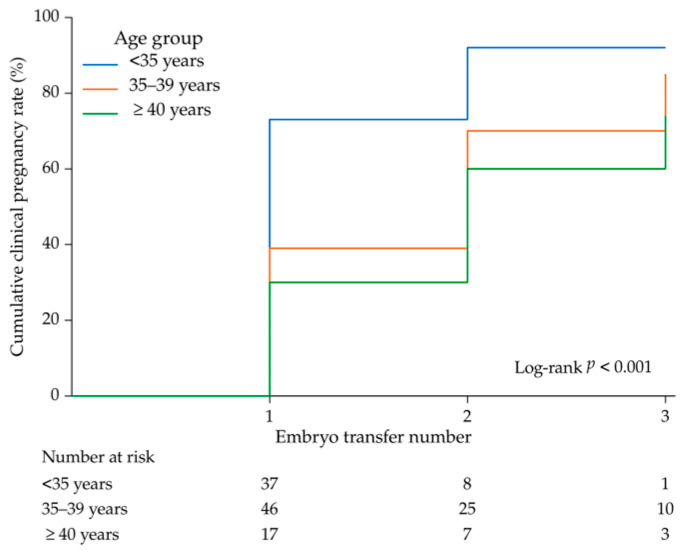
Kaplan–Meier curves of cumulative pregnancy rates up to three embryo transfers stratified by age group. Patients were categorized into three age groups (≤34, 35–39, and ≥40 years). Clinical pregnancy was defined as the event, and the number of embryo transfers was used as the time scale. Differences among age groups were evaluated using the log-rank test.

The observed CCPR, considering treatment discontinuation as non-pregnancy, was 79.0%. The age-stratified CCPR was 91.9% in women aged ≤34 years, 78.3% in women aged 35–39 years, and 52.9% in women aged ≥40 years. Overall, 13 patients (13.0%) discontinued treatment before pregnancy, and the discontinuation rate increased with age (Figure 2).

The reasons for treatment discontinuation are summarized in Table S1 (available at Zenodo). Most discontinuations were related to oocyte depletion, particularly in women aged ≥40 years.

#### 3.4.2. Kaplan–Meier-Estimated Cumulative Pregnancy Rate

When treatment discontinuation was treated as censoring, the KM-estimated cumulative pregnancy rate at ET3 was 87.4%. The absolute difference between the observed CCPR and KM estimates increased with age (+2.0% in ≤34 years, +7.1% in 35–39 years, and +20.2% in ≥40 years), reflecting the higher discontinuation rate among older women (Table 3).

Log-rank testing demonstrated significant differences among the age groups (χ^2^ (2) = 14.59, *p* < 0.001). In the Cox proportional hazards analysis (reference: ≤34 years), women aged 35–39 years (HR 0.585, 95% CI 0.360–0.949, *p* = 0.030) and ≥40 years (HR 0.443, 95% CI 0.211–0.929, *p* = 0.031) had significantly lower probabilities of achieving pregnancy (Table 4).

Age-stratified embryo-related variables showed no significant differences in the rate of cycles with at least one good-quality blastocyst transferred (*p* = 0.766) or in the blastocyst transfer rate (*p* = 0.091). However, anti-Müllerian hormone levels were significantly lower (*p* = 0.024), and dropout rates were significantly higher (*p* = 0.008) in older women (Table 5).

### 3.5. Integrated Results: Patient-Level and Cycle-Level Analyses

Multivariate logistic regression analysis was performed to identify independent predictors of cumulative pregnancy within three ET cycles at the patient level (Table 6). Increased age was significantly associated with a lower cumulative pregnancy rate (OR 0.62, 95% CI 0.49–0.80, *p* < 0.001). Endometriosis was also identified as an independent negative predictor (OR 0.07, 95% CI 0.01–0.45; *p* = 0.004).

The preoperative number of endometrial polyps, categorized into three groups (1, 2–4, and ≥5), was associated with cumulative pregnancy rates after polypectomy. Compared with patients with a single polyp, those with 2–4 polyps had higher odds of cumulative pregnancy (OR 8.79, 95% CI 1.18–65.56, *p* = 0.034), and those with ≥5 polyps had higher odds (OR 18.02, 95% CI 2.64–123.02, *p* = 0.003).

BMI (OR 0.89, 95% CI 0.74–1.06, *p* = 0.16), CE score (OR 0.97, 95% CI 0.37–2.52, *p* = 0.94), and male infertility (OR 0.28, 95% CI 0.063–1.27, *p* = 0.099) were not independently associated with cumulative pregnancy after adjustment.

To complement the patient-level findings, we restructured the cycle-level analysis to reduce conceptual overlap among embryo-related variables. In the primary all-cycle GEE model, which included maternal age, embryo stage, number of embryos transferred, and endometrial thickness, increasing age remained significantly associated with reduced odds of clinical pregnancy per cycle (OR 0.84, 95% CI 0.76–0.94, *p =* 0.002). Blastocyst transfer was independently associated with higher odds of pregnancy compared with cleavage-stage transfer (OR 9.13, 95% CI 1.27–65.87, *p =* 0.028). The number of embryos transferred (OR 0.74, 95% CI 0.37–1.48, *p =* 0.395) and endometrial thickness (OR 1.20, 95% CI 1.00–1.45, *p =* 0.056) were not significantly associated with pregnancy per cycle (Table 7).

In a sensitivity analysis restricted to blastocyst transfer cycles using a GEE model accounting for clustering by patient (Table S2 available at Zenodo), the direction and significance of the age effect remained unchanged (OR 0.86, 95% CI 0.78–0.95, *p =* 0.004). The presence of at least one good-quality blastocyst was not independently associated with pregnancy per cycle after adjustment for number of embryos transferred and endometrial thickness (OR 1.47, 95% CI 0.73–2.97, *p =* 0.282). The number of embryos transferred (OR 0.74, 95% CI 0.36–1.49, *p =* 0.394) and endometrial thickness (OR 1.19, 95% CI 0.99–1.43, *p =* 0.059) were also not significantly associated with pregnancy per cycle. These findings support the robustness of the age effect and indicate that the main cycle-level results were not driven by structural overlap between embryo stage and blastocyst quality variables.

### 3.6. Exploratory Pre- and Post-Polypectomy Comparison in Patients with Prior Failed Embryo Transfer

Among the 30 patients with previously unsuccessful ET before polypectomy, no clinical pregnancies were recorded across 99 preoperative ET cycles. After polypectomy, 21 of the 30 patients (70.0%) achieved clinical pregnancy within up to three subsequent ET cycles. Among the 18 patients who met the criteria for recurrent implantation failure, 12 (66.7%) achieved pregnancy after polypectomy. These descriptive findings are presented as exploratory observations and should not be interpreted as confirmatory evidence of the treatment effect.

The available baseline and preoperative ET characteristics of this subgroup are summarized in Table S3 (available at Zenodo).

### 3.7. Cumulative Live Birth and Miscarriage Outcomes

Detailed age-stratified reproductive outcomes, including the number of clinical pregnancies, live births, and miscarriages and the corresponding live birth and miscarriage rates among clinically pregnant women, are summarized in Table 8. Among the 100 patients who initiated ET, the CLBR within three ET cycles was 65.0% (65/100). Among the 79 patients who achieved clinical pregnancy, 65 delivered, seven experienced miscarriage, and seven had ongoing pregnancies at the time of analysis. The live birth rate among clinically pregnant women was 82.3% (65/79), and the miscarriage rate was 8.9% (7/79).

Age-stratified analysis showed a progressive decline in live birth rates with advancing maternal age. The live birth rate among clinically pregnant women was 94.1% in women aged ≤34 years, 77.8% in women aged 35–39 years, and 55.6% in women aged ≥40 years. Conversely, miscarriage rates increased with maternal age and reached 44.4% among women aged ≥40 years.

These findings indicate that while cumulative clinical pregnancy remained substantial even in older women, progression to live birth decreased markedly with advancing maternal age.

## 4. Discussion

This retrospective single-center study evaluated cumulative reproductive outcomes after hysteroscopic endometrial polypectomy using HTRS in infertile women undergoing up to three ET cycles. By integrating patient-level cumulative analyses with complementary statistical approaches, including crude CCPR, KM estimation, and secondary cycle-level modeling, this study describes cumulative reproductive outcomes within three ET cycles, suggests that maternal age is a key determinant across analytical levels, and provides an integrated evaluation of cumulative and cycle-level reproductive outcomes following HTRS polypectomy.

### 4.1. Cumulative Pregnancy Outcomes After HTRS Polypectomy

This study describes the cumulative reproductive outcomes of women with infertility who underwent hysteroscopic endometrial polypectomy using HTRS and subsequently underwent ET. Within three ET cycles, the observed CCPR was 79.0%, whereas the KM-estimated cumulative pregnancy probability reached 87.4%.

Because this study did not include a non-surgical comparison group, the findings should be interpreted as descriptive rather than causal. The favorable outcomes observed across successive ET cycles may reflect the cumulative probability inherent in repeated transfer attempts and cannot be specifically attributed to correction of intrauterine pathology in the absence of a non-surgical comparison group.

Endometrial polyps have been associated with impaired implantation through mechanical distortion, altered endometrial receptivity, inflammatory microenvironment changes, and dysregulated cytokine expression [3,4,5]. Previous studies have suggested that hysteroscopic removal of polyps may be associated with improved pregnancy outcomes in both intrauterine insemination and in vitro fertilization populations [6,13,16]. The cumulative reproductive outcomes observed in the present cohort are broadly consistent with these reports.

All procedures in the present study were performed under direct hysteroscopic visualization using a non-thermal mechanical resection system. Although preservation of the endometrial basal layer is theoretically advantageous, this study was not designed to compare surgical techniques. Therefore, no conclusions can be drawn regarding the superiority of HTRS over other hysteroscopic methods.

### 4.2. Maternal Age as the Primary Determinant of Cumulative Outcome

Maternal age was the most consistent determinant of reproductive outcomes. Age was independently associated with cumulative pregnancies in the primary patient-level analysis and remained a consistent predictor in the secondary cycle-level GEE analysis, indicating both sustained and per-transfer effects.

Although crude CCPR markedly declined in women aged ≥40 years, KM estimation demonstrated that part of this difference was attributable to the higher treatment discontinuation rates in older women. The widening discrepancy between CCPR and KM estimates with advancing age underscores the importance of accounting for treatment discontinuation when interpreting cumulative reproductive outcomes.

Interestingly, morphological embryo quality did not differ significantly across age groups. The rate of cycles with at least one good-quality blastocyst transferred and the blastocyst transfer rate were comparable across age groups. These findings suggest that the lower cumulative pregnancy rates in older women cannot be solely explained by differences in morphological grading.

PGT-A was not performed in this cohort; therefore, embryonic chromosomal competence could not be assessed directly, and the contribution of age-related aneuploidy to the reduced cumulative pregnancy and increased miscarriage rates could not be quantified. Nevertheless, the persistence of age as an independent predictor in the multivariate and survival analyses supports the interpretation that biological reproductive aging, rather than morphological embryo grading alone, plays a central role in cumulative outcomes.

From a diagnostic perspective, distinguishing cumulative (patient-level) prognosis from per-cycle success probability may improve individualized counseling and risk stratification in women of advanced maternal age. The integration of patient age, ovarian reserve markers, and treatment continuation behavior is essential for accurate prognostic interpretation.

Taken together, these findings indicate that reduced cumulative pregnancy in women aged ≥40 years is primarily attributable to reproductive aging and higher discontinuation rates rather than inferior morphological embryo quality alone.

Discontinuation was more frequent among older women and was most strongly related to oocyte depletion (Supplementary Table S1). Discontinuation may reflect diminished reproductive potential, and censoring may not be completely informative. Therefore, KM estimates should be interpreted alongside the observed CCPR when evaluating cumulative pregnancy outcomes.

### 4.3. Hierarchical Structure of Determinants: Primary and Secondary Analyses

An important conceptual contribution of this study is the distinction between determinants that operate at different hierarchical levels.

In the primary patient-level analysis, maternal age was associated with cumulative reproductive potential across three ET cycles. In addition, the preoperative number of endometrial polyps was associated with cumulative pregnancy after polypectomy.

This finding should not be interpreted as evidence that endometrial polyps improve fertility. Because all patients in this cohort underwent hysteroscopic polypectomy before ET, the observed association reflects the relationship between baseline polyp burden and post-polypectomy reproductive outcomes. One possible explanation is that patients with more extensive but surgically correctable intracavitary pathology may show more favorable post-polypectomy reproductive outcomes; however, the CIs were wide and the single-polyp reference group was small. Therefore, this observation should be regarded as exploratory and interpreted with caution. Because patients with a CE score ≥2 received antibiotic treatment before ET and repeat biopsy was not routinely performed, the independent effect of antibiotic therapy could not be separated from that of polypectomy in this retrospective study design. Although CE score was not significantly associated with cumulative pregnancy in the adjusted analysis, residual confounding related to endometrial treatment cannot be excluded.

In the secondary cycle-level analysis, embryo developmental stage was more strongly associated with the probability of pregnancy per transfer. Together, these findings suggest a hierarchical structure of determinants: patient-level biological factors establish the overall reproductive potential, whereas embryo-level characteristics influence the success of individual transfer attempts.

This framework may assist clinicians in distinguishing factors that determine cumulative prognosis from those that primarily affect per-cycle optimization strategies.

Notably, the magnitude of the age effect differed between patient-level logistic regression and cycle-level GEE analyses. The OR for age was lower in the cycle-level model (0.84 per year) than in the patient-level cumulative analysis (0.63 per year). This difference was expected because the two models addressed different clinical questions and units of analysis. The patient-level model evaluated the probability of achieving at least one pregnancy across up to three ET attempts, thereby incorporating both the biological reproductive potential and the cumulative effect of repeated transfers. In contrast, the cycle-level GEE model estimated the probability of pregnancy in an individual transfer while accounting for within-patient correlation. Consequently, the cycle-level estimate reflects the per-transfer influence of age, whereas the patient-level estimate captures the compounded effect of age across multiple treatment attempts. The age effect was consistent across the patient-level and cycle-level analyses.

### 4.4. Live Birth Outcomes and Post-Implantation Attrition

Although the CCPR was substantial, cumulative live birth outcomes showed a more pronounced age-dependent decline. Among women who achieved clinical pregnancy, the miscarriage rate was markedly higher among those aged ≥40 years than among those in younger age groups.

These findings suggest that age-related embryonic chromosomal instability remains an important limitation for progression from clinical pregnancy to live birth, even after polypectomy. The absence of PGT-A in this cohort precluded the direct evaluation of embryonic chromosomal status; however, the observed increase in miscarriage with age is biologically consistent with the known increase in aneuploidy rates at advanced maternal age [17,18].

These findings highlight an important distinction: cumulative clinical pregnancy does not necessarily translate to cumulative live births, particularly in older women. Consequently, CLBR may be a clinically meaningful endpoint for patient counseling [15].

### 4.5. Interpretation of Exploratory Pre- and Post-Polypectomy Findings

An exploratory pre- and post-polypectomy comparison in patients with prior unsuccessful ET showed an apparent difference between the preoperative and postoperative periods. However, this observation should be regarded as hypothesis-generating rather than confirmatory. The comparison was uncontrolled, and regression to the mean could not be excluded. In addition, important factors may have changed over time, including embryo characteristics, laboratory conditions, treatment protocols, uterine environment, and patient selection.

Furthermore, detailed standardized preoperative cycle-level characteristics were not fully available for direct comparison. Therefore, although the postoperative pregnancy rate in this subgroup may be clinically suggestive, it does not provide causal evidence that polypectomy was responsible for the observed pregnancies. The striking absence of preoperative pregnancies (0/99 ET cycles) should likewise be interpreted cautiously in light of these limitations.

### 4.6. Selection Bias

The exclusion of patients who conceived before ET may have introduced a selection bias. Women who achieved spontaneous pregnancy or pregnancy following intrauterine insemination after polypectomy may have had relatively preserved fertility potential, whereas women who discontinued treatment before ET may have had different prognostic characteristics. Consequently, our study findings should be interpreted specifically in a population of patients who underwent ET after HTRS-assisted hysteroscopic polypectomy and may not be generalizable to all women who undergo hysteroscopic polypectomy.

### 4.7. Strengths and Limitations

This study has some limitations. First, the generalizability of the findings may be limited because of the retrospective, single-center design. Second, the sample size was modest, which may have reduced statistical power and contributed to imprecise estimates for some cycle-level predictors. Third, heterogeneity in ET practice, including embryo stage, number of embryos transferred, and endometrial preparation protocols, may have introduced residual confounding, despite multivariable adjustment. Fourth, the exclusion of patients who did not initiate ET after hysteroscopic polypectomy may have introduced selection bias, and the findings should therefore be interpreted specifically in women who underwent ET after HTRS-assisted hysteroscopic polypectomy. Finally, this study did not include any non-surgical comparison group; therefore, the observed associations should be interpreted as descriptive rather than as evidence of a causal treatment effect.

Despite these limitations, this study also has several strengths. The analysis evaluated cumulative reproductive outcomes across up to three ET cycles, providing a descriptive assessment of reproductive outcomes after HTRS-assisted hysteroscopic polypectomy among infertile women who proceeded to ET. In addition, both patient-level and cycle-level statistical approaches were applied, allowing for complementary evaluation of cumulative pregnancy probability and per-cycle pregnancy determinants. This approach may be useful for distinguishing factors associated with overall cumulative prognosis from those associated with the probability of pregnancy in an individual ET cycle.

From a practical and educational perspective, these findings may help inform counseling for infertile women who proceed to ET after HTRS-assisted hysteroscopic polypectomy by emphasizing age-stratified cumulative outcomes, treatment discontinuation, and the distinction between cumulative and per-cycle pregnancy probabilities.

Future prospective, multicenter studies with larger cohorts and appropriate comparison groups, including women managed without polypectomy and, where feasible, women treated using other hysteroscopic techniques, are needed. Further studies incorporating standardized ET protocols, longer follow-up to live birth, detailed embryo-quality or chromosomal information, and reasons for treatment discontinuation may help clarify the uterine, embryonic, and patient-level factors associated with reproductive outcomes after hysteroscopic polypectomy.

## 5. Conclusions

This study describes cumulative reproductive outcomes within three ET cycles among infertile women who underwent ET after HTRS-assisted hysteroscopic endometrial polypectomy. Maternal age was consistently associated with both cumulative and per-cycle pregnancy probabilities across analytical approaches.

These findings may support age-stratified counseling, particularly when treatment discontinuation is considered in interpreting cumulative reproductive outcomes.

Given the observational design and absence of a non-surgical comparison group, the findings should be interpreted as descriptive associations rather than evidence of a causal treatment effect. Further prospective studies with appropriate comparison groups are warranted.

## Figures and Tables

**Table 1:** Baseline characteristics according to clinical pregnancy within three embryo transfers.

Characteristics	Pregnancy Group (*n* = 79)	Non-Pregnancy Group (*n* = 21)	*p*-Value
Age (years), mean ± SD	35.08 ± 3.45	38.28 ± 3.19	<0.001 *
Gravidity, mean ± SD	0.56 ± 0.91	0.66 ± 0.85	0.66
Parity, mean ± SD	0.20 ± 0.43	0.28 ± 0.46	0.44
BMI (kg/m^2^), mean ± SD	22.10 ± 3.08	23.95 ± 4.23	0.03 *
AMH (ng/mL), mean ± SD	3.24 ± 4.07	1.86 ± 1.87	0.13
Preoperative ET cycles, mean ± SD	0.93 ± 2.01	1.23 ± 1.81	0.53
Number of preoperative endometrial polyps, *n* (%)			0.05
1	7 (8.9)	5 (23.8)	
2–4	21 (26.6)	8 (38.1)	
≥5	51 (64.6)	8 (38.1)	
CE score, *n* (%)			0.96
0	11 (13.9)	2 (9.5)	
1	54 (68.4)	15 (71.4)	
2	9 (11.4)	3 (14.3)	
3	3 (3.8)	1 (4.8)	
Untested	2 (2.5)	0 (0.0)	
Infertility causes †, *n* (%)			
Endometriosis	8 (10.1)	6 (28.6)	0.069
Tubal factor	5 (6.3)	2 (9.5)	0.635
Ovulatory dysfunction	8 (10.1)	0 (0.0)	0.198
Unexplained	20 (25.3)	4 (19.0)	0.775
Male infertility	51 (64.6)	16 (76.2)	0.435

The non-pregnancy group included patients who did not achieve pregnancy within three ET cycles, including those who discontinued treatment before completing the three cycles. Data are presented as mean ± SD or *n* (%). Continuous variables were compared using Student’s *t*-test, and categorical variables were analyzed using the chi-square test or Fisher’s exact test, as appropriate. * *p* < 0.05 was considered statistically significant. † Multiple infertility causes could be assigned to one patient. BMI, body mass index; AMH, anti-Müllerian hormone; CE, chronic endometritis; SD, standard deviation.

**Table 2:** Embryo transfer characteristics (cycle-based analysis) up to three embryo transfer cycles.

Characteristics	Pregnancy Group (*n* = 79)	Non-Pregnancy Group (*n* = 21)	*p*-Value
Total ET cycles, *n*	121	41	—
Embryos transferred per cycle, mean ± SD	1.23 ± 0.42	1.41 ± 0.50	0.02 *
Single embryo transfer cycles, *n* (%)	93 (76.9)	24 (58.5)	0.02 *
Double embryo transfer cycles, *n* (%)	28 (23.1)	17 (41.5)	
Endometrial thickness (mm), mean ± SD	11.11 ± 2.05	10.61 ± 1.76	0.16
Fresh ET cycles, *n* (%)	4 (3.3)	3 (7.3)	0.44
Frozen–thawed ET cycles, *n* (%)	117 (96.7)	38 (92.7)	
Hormone replacement cycles, *n* (%)	61 (50.4)	22 (53.7)	
Natural cycles, *n* (%)	56 (46.3)	16 (39.0)	
Cleavage-stage transfer cycles, *n* (%)	5 (4.1)	7 (17.1)	0.01 *
Blastocyst transfer cycles, *n* (%)	116 (95.9)	34 (82.9)	
Cycles with ≥1 good-quality blastocyst transferred *, *n* (%)	90 (74.4)	17 (41.5)	0.004 *

Data are presented per embryo transfer cycle. Pregnancy was defined at the patient level. Defined as cycles in which at least one transferred blastocyst was graded as AA, AB, or BA. In double ET cycles, classification was based on the higher-grade embryo transferred. Data are presented as mean ± SD or number of cycles (*n*). Continuous variables were compared using Student’s *t*-test, and categorical variables were analyzed using the chi-square test or Fisher’s exact test, as appropriate. * *p* < 0.05 was considered statistically significant. SD, standard deviation.

**Table 3:** Observed cumulative clinical pregnancy rate (CCPR) and Kaplan–Meier-estimated cumulative pregnancy rate (up to ET3).

Age Group	*n* (ET1 Start)	Pregnancies (≤ET3)	CCPR (%)	KM Estimate at ET3 (%)	Absolute Difference (%)
Overall	100	79	79.0	87.4	+8.4
≤34 years	37	34	91.9	93.9	+2.0
35–39 years	46	36	78.3	85.4	+7.1
≥40 years	17	9	52.9	73.1	+20.2

CCPR was calculated as the proportion of patients who achieved at least one clinical pregnancy within three ET cycles among all patients who initiated ET1. Patients who discontinued treatment without pregnancy were considered non-pregnant in the CCPR calculations. Kaplan–Meier estimates were calculated by treating pregnancy as an event and treatment discontinuation as right censoring. The Kaplan–Meier method assumes non-informative censoring, meaning that censored patients are assumed to have the same future probability of pregnancy as those remaining under observation. CCPR, cumulative clinical pregnancy rate; ET, embryo transfer; KM, Kaplan–Meier.

**Table 4:** Log-rank test and Cox proportional hazards analysis of cumulative pregnancy according to age group.

Analysis	Comparison	Test Statistic/HR	95% CI	df	*p*-Value
Log-rank test	Global (3 groups)	χ^2^ = 14.59	—	2	<0.001 *
	≤34 vs. 35–39 years	Z = 3.21	—	1	0.001 *
	≤34 vs. ≥40 years	Z = 3.18	—	1	0.001 *
	35–39 vs. ≥40 years	Z = 0.94	—	1	0.348
Cox model *	35–39 vs. ≤34 years	HR = 0.585	0.360–0.949	—	0.030 *
	≥40 vs. ≤34 years	HR = 0.443	0.211–0.929	—	0.031 *

Kaplan–Meier survival curves were compared using the log-rank test. Cox proportional hazards regression was performed to estimate hazard ratios (HRs) for achieving pregnancy. The ≤34 years group was used as the reference category in the Cox model. HR < 1 indicates a reduced likelihood of achieving pregnancy compared with the reference group. * *p* < 0.05 was considered statistically significant.

**Table 5:** Age-stratified embryo quality, ovarian reserve, and dropout rates up to three embryo-transfer cycles.

Variable	≤34 Years	35–39 Years	≥40 Years	*p*-Value
Blastocyst transfer rate	97.9%	92.7%	84.8%	0.091
Rate of cycles with ≥1 good-quality blastocyst transferred	76.6%	72.0%	69.7%	0.766
AMH (mean ± SD)	4.14 ± 5.68	2.60 ± 1.55	1.33 ± 1.30	0.024
Dropout rate	5.4%	10.9%	35.3%	0.008

Data are presented as percentages or mean ± standard deviation (SD). Blastocyst transfer rate and the rate of cycles with at least one good-quality blastocyst transferred were calculated from all ET cycles performed up to ET3 within each age group. In double ET cycles, blastocyst quality was assigned according to the higher-grade embryo transferred; cycles were classified as positive when at least one transferred blastocyst was graded as AA, AB, or BA. Cleavage-stage transfer cycles were classified as having no good-quality blastocyst transferred. Dropout was defined as treatment discontinuation before the completion of three ET cycles without achieving pregnancy. Comparisons among age groups were performed using the chi-square test for categorical variables and one-way ANOVA for continuous variables. Statistical significance was set at *p* < 0.05. AMH, anti-Müllerian hormone.

**Table 6:** Multivariable logistic regression analysis for clinical pregnancy within three embryo transfer cycles (patient-level analysis).

Variable	OR	95% CI	*p*-Value
Age (per year increase)	0.62	0.49–0.80	<0.001 *
BMI	0.89	0.74–1.06	0.16
Number of preoperative endometrial polyps (reference = 1 polyp)			
2–4 vs. 1	8.79	1.18–65.56	0.034 *
≥5 vs. 1	18.02	2.64–123.02	0.003 *
CE score (per unit increase)	0.97	0.37–2.52	0.94
Endometriosis	0.07	0.01–0.45	0.004 *
Male infertility	0.28	0.06–1.27	0.09

All analyses were performed at the patient level. Cumulative pregnancy was defined as pregnancy achieved within three embryo transfer (ET) cycles per patient. Age, BMI, and CE scores were modeled as continuous variables (per-unit increase). The number of endometrial polyps was categorized into three groups (1, 2–4, and ≥5), with the single-polyp group serving as the reference category. Endometriosis and male infertility were considered binary variables. * *p* < 0.05 was considered statistically significant. BMI, body mass index; CE, chronic endometritis; OR, odds ratio; CI, confidence interval.

**Table 7:** GEE (all-cycle) analysis for pregnancy in each embryo transfer cycle (clustered by patient).

Variable	OR	95% CI	*p*-Value
Age (per year)	0.84	0.76–0.94	0.002
Embryo stage (blastocyst vs. Cleavage-stage)	9.13	1.27–65.87	0.028
Number of embryos transferred	0.74	0.37–1.48	0.395
Endometrial thickness (per mm)	1.20	1.00–1.45	0.056

Analyses were conducted at the cycle level (one row per embryo transfer cycle) and clustered by patient ID to account for within-patient correlations. A binomial distribution with a logit link and an exchangeable working correlation structure was used, and robust (sandwich) standard errors were applied. The primary all-cycle model included maternal age, embryo stage, number of embryos transferred, and endometrial thickness. Because blastocyst quality is only defined among blastocyst transfer cycles, the presence of at least one good-quality blastocyst transferred was evaluated separately in a sensitivity analysis restricted to blastocyst transfer cycles (Table S2). Odds ratios for continuous variables represent the change per-unit increase. Statistical significance was set at *p* < 0.05. OR, odds ratio; CI, confidence interval; GEE, generalized estimating equations.

**Table 8:** Age-stratified reproductive outcomes within three embryo transfer cycles.

Age Group (Years)	Total Patients (*n*)	Clinical Pregnancies (*n*)	Live Births (*n*)	Miscarriages (*n*)	Live Birth Rate (%) ^1^	Miscarriage Rate (%) ^1^
Total	100	79	65	7	82.3	8.9
≤34	37	34	32	0	94.1	0.0
35–39	46	36	28	3	77.8	8.3
≥40	17	9	5	4	55.6	44.4

^1^ Live birth and miscarriage rates were calculated for women who achieved clinical pregnancy.

## Data Availability

Supplementary Data are available in Zenodo at https://doi.org/10.5281/zenodo.19652688 (Published 15 March 2026). Additional data are available from the corresponding author upon reasonable request and are not publicly available due to privacy and ethical restrictions.

## Supplementary Materials

The following supporting information can be downloaded at: https://www.mdpi.com/article/10.3390/diagnostics16091386/s1, Table S1, Reasons for treatment discontinuation before completion of three embryo transfer cycles, stratified by age group and timing of discontinuation; Table S2, GEE analysis restricted to blastocyst transfer cycles for clinical pregnancy per cycle (clustered by patient); and Table S3, Descriptive characteristics of the subgroup of patients with prior failed embryo transfer before polypectomy and available preoperative ET cycle data (https://doi.org/10.5281/zenodo.19652688, Published 15 March 2026).

## Abbreviations

The following abbreviations are used in this manuscript:

AMH	anti-Müllerian hormone
BMI	body mass index
CCPR	cumulative clinical pregnancy rate
CE	chronic endometritis
CLBR	cumulative live birth rate
ET	embryo transfer
GEE	generalized estimating equations
HR	hazard ratio
HTRS	hysteroscopic tissue removal system
KM	Kaplan–Meier
OR	odds ratio
PGT-A	preimplantation genetic testing for aneuploidy
