# Supplementary material for: Cumulative Reproductive Outcomes Across Three Embryo Transfer Cycles After Hysteroscopic Endometrial Polypectomy Using a Tissue Removal System in Infertile Women: A Single-Center Retrospective Cohort Study
Source: Diagnostics (Basel). 2026 May 2;16(9):1386. doi: 10.3390/diagnostics16091386 (PMC13163227; doi:10.3390/diagnostics16091386)
Supplement: Supplementary file 1 [file diagnostics-16-01386-s001.zip › diagnostics-4242320-Tables S1-S3.pdf]

Table S1. Reasons for treatment discontinuation before completion of three embryo transfer cycles, stratified by age group and timing of discontinuation.

Age group (years)	After ET1	After ET2
≤34	Oocyte depletion (n = 1)	Patient-requested discontinuation (n = 1)
35–39	Oocyte depletion (n = 2);<br>Patient-requested discontinuation (n = 1)	Oocyte depletion (n = 2)
≥40	Transfer to another clinic (n = 1);<br>Oocyte depletion (n = 4)	Oocyte depletion (n = 2)

Discontinuation was defined as the cessation of infertility treatment before completing three embryo transfer (ET) cycles without achieving clinical pregnancy. The reasons for discontinuation were extracted from clinical records. Oocyte depletion refers to cases in which no further embryos are available for transfer.

Table S2. GEE analysis restricted to blastocyst transfer cycles for clinical pregnancy per cycle (clustered by patient).

Variable	OR	95% CI	p-value
Age (per year)	0.86	0.78–0.95	0.004
Number of embryos transferred	0.74	0.36–1.49	0.394
Presence of ≥1 good-quality blastocyst transferred	1.47	0.73–2.97	0.282
Endometrial thickness (per mm)	1.19	0.99–1.43	0.059

This sensitivity analysis was restricted to blastocyst transfer cycles only. Analyses were conducted at the cycle level and clustered by patient ID to account for within-patient correlations. A binomial distribution with a logit link and an exchangeable working correlation structure was used, and robust (sandwich) standard error was applied. In double embryo transfer cycles, the variable “presence of ≥1 good-quality blastocyst transferred” was coded according to the higher-grade embryo transferred; cycles were classified as positive when at least one transferred blastocyst was graded as AA, AB, or BA. Odds ratios for continuous variables represent the change per unit increase. Statistical significance was set at $p < 0.05$.

OR, odds ratio; CI, confidence interval; GEE, generalized estimating equations.

Table S3. Descriptive characteristics of the subgroup of patients with prior failed embryo transfer before polypectomy and available preoperative ET cycle data.

Characteristic	Value
**Patient-level characteristics (n = 30)**	
Age at surgery (years), mean ± SD	35.8 ± 4.0
Infertility duration (months), mean ± SD	57.9 ± 30.5
Failed ET cycles before polypectomy, mean ± SD	3.3 ± 2.3
Failed ET cycles before polypectomy, median (IQR)	3 (2–4)
Patients with ≥3 failed ET cycles before polypectomy, n (%)	18 (60.0)
**Available preoperative ET cycle characteristics (n = 99 cycles)**	
Cleavage-stage transfer cycles, n (%)	11 (11.1)
Blastocyst transfer cycles, n (%)	88 (88.9)
Single embryo transfer cycles, n (%)	77 (77.8)
Double embryo transfer cycles, n (%)	22 (22.2)
Fresh ET cycles, n (%)	6 (6.1)
Frozen–thawed hormone replacement cycles, n (%)	51 (51.5)
Frozen–thawed natural cycles, n (%)	36 (36.4)
Protocol unavailable, n (%)	6 (6.1)

Values are presented descriptively to improve transparency. Detailed preoperative cycle-level records were available for 99 of the 100 preoperative ET cycles. ET, embryo transfer; IQR, interquartile range.
